# The Relation Between Trait Anger and Impulse Control in Forensic Psychiatric Patients: An EEG Study

**DOI:** 10.1007/s10484-018-9393-5

**Published:** 2018-05-24

**Authors:** Marien Lievaart, Frederik M. van der Veen, Jorg Huijding, Johannes E. Hovens, Ingmar H. A. Franken

**Affiliations:** 1 Institute of Psychology, Erasmus University Rotterdam, P. O. Box 1738, 3000 DR Rotterdam, The Netherlands; 2 Utrecht University, Utrecht, The Netherlands; 3 Antes Mental Health Care, Rotterdam, The Netherlands

**Keywords:** EEG, Trait anger, Error related negativity, Inhibitory control, Impulsivity, N2, P3

## Abstract

Inhibitory control is considered to be one of the key factors in explaining individual differences in trait anger and reactive aggression. Yet, only a few studies have assessed electroencephalographic (EEG) activity with respect to response inhibition in high trait anger individuals. The main goal of this study was therefore to investigate whether individual differences in trait anger in forensic psychiatric patients are associated with individual differences in anger-primed inhibitory control using behavioral and electrophysiological measures of response inhibition. Thirty-eight forensic psychiatric patients who had a medium to high risk of recidivism of violent and/or non-violent behaviors performed an affective Go/NoGo task while EEG was recorded. On the behavioral level, we found higher scores on trait anger to be accompanied by lower accuracy on NoGo trials, especially when anger was primed. With respect to the physiological data we found, as expected, a significant inverse relation between trait anger and the error related negativity amplitudes. Contrary to expectation, trait anger was not related to the stimulus-locked event related potentials (i.e., N2/P3). The results of this study support the notion that in a forensic population trait anger is inversely related to impulse control, particularly in hostile contexts. Moreover, our data suggest that higher scores on trait anger are associated with deficits in automatic error-processing which may contribute the continuation of impulsive angry behaviors despite their negative consequences.

## Introduction

Anger is seen as a negative approach-related emotion (Carver and Harmon-Jones 2009) that typically entails the action tendency to counter or redress perceived wrongdoings (Fernandez 2013). Anger can occasionally lead to positive outcomes, such as eliciting compliance and co-operation from others (e.g., van Doorn et al. 2014), gaining a stronger sense of control, and signaling the desire to change the problematic nature of a situation (Fischer and Roseman 2007). Moreover, diary studies show that people experience anger regularly and mostly solve their anger in nonaggressive, prosocial ways (Averill 1983; Kassinove et al. 1997). Anger can thus be considered as a basic and adaptive emotion which may not necessarily be problematic, if regulated properly. However, anger can also lead to less desirable outcomes. For instance, uncontrolled anger can make people say hurtful things they later regret and serves as a proximate cause of violent offending and aggressive behavior (Novaco 2011). Anger becomes especially dysfunctional when regulated improperly or when it becomes part of someone’s personality, such that it starts to interfere with daily functioning and becomes excessive in its frequency, intensity, duration, and expression (DiGiuseppe and Tafrate 2010; Spielberger 1999). For instance, high trait anger is a robust predictor of aggressive behavior (Bettencourt et al. 2006; Tafrate et al. 2002), and is associated with domestic violence (Barbour et al. 1998), poorer psychosocial functioning (McDermut et al. 2009), and interpersonal problems (Baron et al. 2006). Given the negative outcomes associated with anger combined with society’s disapproval of angry disruptions (Stearns and Stearns 1989), people are frequently motivated to regulate and control their angry impulses (Tice and Baumeister 1993). Accordingly, brain regions corresponding with controlled top-down emotion regulation and inhibitory control, such as the lateral and medial prefrontal cortex, show increased activity after being provoked (Denson et al. 2009; Krämer et al. 2008). Following this, it seems likely that anger-prone individuals lack the ability to override these angry impulses and control themselves (Davidson 2000; Denson 2015).

Inhibitory control is considered to be one of the key factors in explaining individual differences in trait anger and reactive aggression (Wilkowski and Robinson 2008b, 2010). More specifically, Wilkowski and Robinson (2008b, 2010) propose in their integrative cognitive model of trait anger and reactive aggression that effortful control, the ability to override dominant cognitive tendencies in favor of subdominant tendencies, mitigates tendencies toward anger and reactive aggression by (a) fostering reappraisal in favor of a non-hostile interpretation, (b) allowing individuals to disengage from hostile ruminative thoughts, and (c) suppressing angry expressions and aggressive behavior tendencies. Of further importance, they propose that effortful control is best conceptualized in terms of a flexible resource that can be exerted in specific contexts, such that individuals low in trait anger recruit sufficient effortful control resources in potential hostile situations in order to keep their cool, whereas high trait anger individuals do not. These assumptions concerning effortful control are in line with studies highlighting the impaired top-down control of the prefrontal cortex over limbic and subcortical regions in aggressive samples (Blair 2012; Davidson 2000; Siever 2008) and with theories proposing that the cognitive control system is particularly activated under circumstances where individuals are motivated to override their automatic response tendencies (Lieberman 2007). In line with the integrative cognitive model of trait anger and reactive aggression, convincing evidence has shown that high trait anger individuals demonstrate lower effortful control on implicit cognitive tasks, especially when anger is primed. For instance, using implicit cognitive tasks, Wilkowski and Robinson (2008a) have shown that low trait anger individuals demonstrated reduced interference compared to high trait anger individuals when primed with aggression-related words. Moreover, individuals high on trait anger showed slower response-inhibition processes for angry expressions and not for neutral expressions on a stop-signal task (Wilkowski 2011). Finally, anger-primed inhibitory control fostered forgiveness in both laboratory and real-life settings subsequently reducing anger and aggressive behavior (Wilkowski et al. 2010).

Despite these important insights, most studies using these implicit cognitive tasks were conducted within healthy subjects high on trait anger. Hence, there is a need for investigating the inverse relation between effortful control and trait anger in forensic patient samples, such as violent offenders. In addition, only a few studies have assessed electroencephalographic (EEG) activity while performing tasks that require effortful control. One study found reduced attentional control and diminished behavioral inhibition in high trait anger offenders on a continuous performance task as evidenced by fewer hits, more false alarm rates, and reduced relative right frontocortical activity compared to a control group (Jaworska et al. 2012). Another study found faster reaction times and a decreased P3 component using difference waveforms (NoGo minus Go) on a Go/NoGo task in high trait anger individuals compared to low trait individuals, suggesting impaired response inhibition (Liu et al. 2014). Notably, they found no differences between low and high trait anger individuals on the N2 component and the P3 component on NoGo trials. As both these studies used affectively neutral tasks, little is known about the time course of anger-primed inhibitory control in high trait anger individuals. The current study was set up with these caveats in mind.

The main goal of the current study was to investigate whether individual differences in trait anger in violent offenders are associated with individual differences in anger-primed inhibitory control using behavioral and electrophysiological measures of response inhibition. For this purpose a novel Go/NoGo task was developed including anger-related pictures and neutral pictures. Go/NoGo tasks are often used measures to measure the ability to inhibit motor responses. Event-related potentials measured in Go/NoGo tasks show increased N2 amplitudes in the frontal region and increased P3 amplitudes in the frontocentral region on NoGo trials compared to Go trials (Falkenstein et al. 1999). Although both components are generally assumed to reflect inhibitory processing, there is still ongoing debate about what these components reflect precisely and which component best reflects inhibitory processing. For instance, the N2 signal has also been proposed to reflect response conflict (Nieuwenhuis et al. 2003), and the signaling of deviant stimulus features (Fox et al. 2000), whereas the P3 has been proposed to reflect the actual inhibitory process itself (Kok et al. 2004; Verleger et al. 2006). As trait anger is negatively related to impulse control, we expected higher scores on trait anger to be associated with reduced N2 and P3 amplitudes. Importantly, in line with the view that high trait anger individuals recruit less cognitive control resources in hostile situations, we expected these associations to be more pronounced for anger-related pictures. On the behavioral level, we expected higher scores on trait anger to be associated with more mistakes on the infrequent NoGo stimuli, in particular for the anger-related pictures.

A second important aspect of cognitive control is error-processing, which allows individuals to select the appropriate behavior, to optimize goal-directed behavior, and to subsequently adapt their behavior (Botvinick et al. 2001; Ridderinkhof et al. 2004). As deficits in error-processing may contribute to the maintenance of impulsive angry behavior despite its negative consequences, we additionally explored whether high scores on trait anger were accompanied by more prominent deficits in error-processing as reflected by reduced amplitudes on the error related negativity (ERN) and the error positivity (Pe). The ERN is a negative-going response-locked component that arises shortly after making commission errors in reaction-time tasks and has traditionally been proposed to reflect online-monitoring of performance either through automatic error detection (Bernstein et al. 1995) or through conflict monitoring (Yeung et al. 2004). The Pe is a positive-going response locked component after commission errors that follows the ERN and appears to reflect the more conscious processing or awareness of the motivational significance of an error (Luijten et al. 2014; Overbeek et al. 2005). Based on previous research showing that externalizing psychopathology is associated with impaired error processing (Hall et al. 2007; Olvet and Hajcak 2008), we expected higher scores on trait anger to be accompanied by reduced ERN and Pe amplitudes.

## Methods and Materials

### Participants

Participants were 40 Dutch speaking psychiatric inpatients from a forensic department of a psychiatric hospital in Belgium. Two participants were discarded from the data analyses as they failed to comprehend the instructions during the experiment, leaving a total of 38 participants (25 males and 13 females). The mean age of this sample was 41 years (*SD* = 9.2) with an age range from 23 to 58 years. The sample consisted of patients with complex psychiatric disorders and various comorbidities who had a medium to high risk of recidivism of violent and/or non-violent behaviors related to their psychopathology, such as theft, arson, robbery, drug and alcohol-related crimes, aggravating assault and battery, domestic violence, and murder. Patients with sexual offenses, primary psychopathy, paraphilias, or exclusive addiction problems are not treated in this forensic hospital and were thus not included in this study. This study was conducted according to the rules of the Helsinki Declaration on informed consent and confidentiality. Informed consent was obtained from all individual participants included in the study. Approval was obtained in writing by the coordinating Ethical Committee of the “Broeders van Liefde” hospitals. Table 1 presents the descriptive statistics for the demographic variables, observed aggressive behavior, and trait anger for the total inpatient forensic psychiatric sample. Notably, the mean Trait Anger score of our sample was in the 52nd percentile of the standardized sample (Hovens et al. 2014).

**Table 1:** Summary statistics for demographic variables, observed aggressive behavior, and trait anger for the forensic psychiatric inpatient sample

	*M*	*SD*
Demographic variables		
Age	41.00	9.20
Males (%)	65.8	
Anger-related measures		
TAS	17.45	4.71
OSAB-Agg	13.11	3.64

*TAS* trait anger scale, *OSAB-Agg* OSAB aggressive behavior

### Instruments

The Dutch version of the State Trait Anger Expression Inventory-2 (STAXI-2; Spielberger 1999; Dutch translation: Hovens et al. 2014) measures the experience, expression, and control of anger. The STAXI-2 contains 57 items that are scored on a 4-point Likert scale (1 = “almost never”, 2 = “sometimes”, 3 = “often”, 4 = “almost always”). The measure comprises six distinct scales, i.e. state anger, trait anger, anger expression-in, anger expression-out, anger control-in, and anger control-out. In the current study we only focused on the trait anger scale (*T-Ang*; range 10–40), which assesses the general tendency to experience more frequent, more intense, and longer episodes of anger. The STAXI-2 has adequate psychometric properties (Hovens et al. 2014; Spielberger 1999).

*The Observation Scale for Aggressive Behavior* (OSAB; Hornsveld et al. 2007) measures observed ward behavior, and includes the subscales irritation/anger, anxiety/gloominess, aggressive behavior, antecedent (to aggressive behavior), Sanction (for aggressive behavior), and Social behavior. The OSAB comprises of 40 items. The staff scores the behavior of inpatients on the ward that has taken place in the preceding week on a four-point Likert scale (from 1 = “no” to 4 = “frequently”). The psychometric properties of the OSAB were evaluated in 220 violent forensic psychiatric inpatients and turned out to be good with sufficient internal consistency, adequate test-retest-reliability, and good inter-rater reliability (Hornsveld et al. 2007).

### Task Paradigm

E-Prime software (Version 2.0; Psychology Software Tools, Pittsburg, PA) was used to administer the tasks. Participants completed all tasks on windows based computers.

### Aggression-Related Go/NoGo Task

An aggression-related version of a Go/NoGo task adapted from Luijten et al. (2011) was used. In this task, participants viewed a series of pictures with an anger-related or neutral content. Each picture was displayed for 200 ms and had a blue or yellow frame. The frame color indicated whether a stimulus was a Go or a NoGo trial. Response assignments were randomized across participants. Each stimulus was followed by a black screen for a randomly varying duration between 1020 and 1220 ms. Participants were explicitly instructed to respond as fast and as accurate as possible to the pictures in Go trials by pressing a button with their index finger, and to withhold their response for the NoGo trials. The task consisted of 112 different anger-related pictures and 112 Neutral pictures selected from the International Affective Picture System (IAPS; Lang et al. 2008) and Google images, that were matched for color, gender and number of people displayed on the pictures. Anger-related pictures displayed scenes of angry and/or fighting people, whereas neutral pictures showed similar scenes of people engaged in non-angry behaviors. Each picture was presented four times, resulting in a total of 896 trials, of which 25% were NoGo and 75% were Go trials. The amount of NoGo trials were equally divided over picture categories (i.e., 112 NoGo trials and 336 Go trials). We used a blocked design with four blocks consisting of 224 trials each. The first two blocks consisted of neutral pictures and the last two blocks consisted of anger-related pictures. This fixed order was chosen to prevent priming and carry-over effects of the anger-related pictures onto the neutral pictures. After each block, participants were given the opportunity to take a short break. The order of Go and NoGo trials was quasi randomized such that at most two NoGo trials were presented consecutively. Before starting the actual task participants performed 23 practice trials involving additional Neutral pictures. Total task duration was about 22 min.

The accuracy rates for Go and NoGo trials as well as the median reaction times (RT) for the Go trials only were used as performance measures for the Go/NoGo task. The reaction time data for the Go trials were calculated after the deletion of incorrect responses and outliers for each individual, i.e., reaction times below 150 ms or above 1500 ms.

### Procedure

Each patient was tested individually in a silent, secluded room. Participants were seated in a comfortable chair, and received a brief general introduction on the EEG measurements and the procedures. Next, electrodes were placed and participants were instructed to sit relaxed and to minimize eye-blinks and body movements during the experiment. Following these instructions, the experimenter explained the first task. Participants first completed an Emotion Recognition task, followed by an emotional Stroop task, and finally the Go/NoGo task during EEG recording. Task instructions were provided before the start of each task. After completion of the three tasks, electrodes were removed and participants completed the STAXI-2 along with additional measures. For sake of brevity only the results for the Go/NoGo Task and the STAXI-2 are reported in this study. After having completed the experiment, participants were thanked for their participation and were given their financial compensation. In total, testing lasted approximately 1 h and 45 min. Finally, the same week in which the participants completed the experiment, the staff was asked to judge the behavior of the patient on the ward in the preceding week using the OSAB.

### EEG Recording and Data Reduction

The electroencephalogram (EEG) was recorded using the BrainAmp MR Plus amplifier system (Brainproducts GmbH) with a 32 channel Acticap with Ag\AgCl electrodes (Fp1, Fp2, F7, F3, F2, F4, F8, FC5, FC1, FC2, FC6, T7, C3, C2, C4, T8, TP9, CP5, CP1, CP2, CP6, TP10, P7, P3, P2, P4, P8, O1, Oz, O2, EOG1, EOG2) according to the international 10/20 system. Two electrodes were placed to an infraorbital and a supraorbital region of the right eye for recording vertical electro-oculogram (VEOG) to correct for eye-movements and blinks. All signals were digitized with a sample rate of 500 Hz and 24-bit A/D conversion, and were filtered offline. BrainVision Recorder (Brain products GmbH, Munich, Germany) was used to process the offline data. Data were offline-referenced to the average reference on two electrodes attached to left and right mastoids. Off-line, EEG and EOG activity was filtered using phase shift-free Butterworth filters (24 dB/ Octave roll off) with a bandpass of .15–30 Hz. The Go/NoGo task EEG data were segmented in epochs from 200 ms before stimulus presentation to 1450 ms after stimulus presentation and 100 ms before the response onset to 600 ms after the response onset. Ocular correction was applied using the Gratton and Coles algorithm (Gratton et al. 1983). The mean 100 ms pre-stimulus period served as baseline. Artefacts were rejected by excluding epochs exceeding ± 100 µV from the average.

For the N2 and P3 components the average ERP waves were calculated for artifact free trials for Neutral Go, Neutral NoGo, Anger-Related Go, and Anger-Related NoGo stimuli separately. Moreover, for calculating these components segments with incorrect responses (miss for Go trials or false alarms for NoGo trials) were excluded from the analyses. The N2 was defined as the average activity within the 225–325 ms time interval after stimulus onset (based on visual inspection) and was studied at the midline frontal electrode site Fz as the N2 is predominantly examined and observed over anterior scalp sites (Falkenstein et al. 1999). The P3 was defined as the average value within the 350–550 ms time interval after stimulus onset (based on visual inspection). The P3 was studied at the midline central electrode site Cz as the P3 in this task is typically observed at midline electrodes (e.g., Rietdijk et al. 2014). The mean number of analyzable Go and NoGo epochs for the N2 and P3 components after removal of the artifacts was 268 and 73 for anger-Related pictures and 275 and 75 for Neutral pictures respectively. Three participants in total were excluded from these ERP analyses; one because of less than 10 artifact free ERP epochs in at least one of the task conditions and two participants as a result of their low performance accuracy on the behavioral data (accuracy rate below 50% on Go trials).

For the ERN and Pe components the average ERP waves were calculated for artifact free trials for correct Go trials (hits for Go trials) and for incorrect NoGo trials (false alarms for NoGo trials). The ERN was defined as the average value in the 25–75 ms range after response onset (e.g., Luijten et al. 2011; Rietdijk et al. 2014). The Pe was defined as the average value in the 200–400 ms range after response onset (Rietdijk et al. 2014). Both the ERN and the Pe were most clearly visible at the midline electrode Cz, therefore these electrodes were chosen in the response-locked analyses (Easdon et al. 2005; Herrmann et al. 2004). To obtain reliable information for the ERN and Pe at least six trials are needed (Olvet and Hajcak 2009; Pontifex et al. 2010). In total, six participants were excluded from these ERP analyses; four participants because of fewer than six artifact free ERP epochs due to too few errors or due to too many artefacts and two participants as a result of their low performance accuracy (< 75% correct on Go trials). The mean number of analyzable epochs for the ERN and Pe components after removal of the artifacts was 598 for correct Go trials (300 and 298 for neutral and anger-Related Go trials respectively) and 47 for incorrect NoGo trials (25 and 27 for Neutral and Anger-Related NoGo trials respectively).

### Statistical Analyses

Data were analyzed using SPSS 22.0. Missing values on the STAXI-2 and the OSAB were replaced with the individual participant’s series mean. A repeated measures analysis of variance (RM-ANOVA with Greenhouse-Geisser adjusted *p* values) was used to analyze the accuracy rates on the Go/NoGo task with Inhibition (Go vs. NoGo) as within subjects variable as well as for the reaction time data on Go trials with picture (anger-related vs. neutral) as within-subjects factor. Further, repeated-measure analyses of covariance (RM-ANCOVA; with Greenhouse-Geisser adjusted *p* values) were used to analyze the accuracy rates on the Go/NoGo task with picture as within-subjects variable and trait anger as covariate. Finally, RM-ANCOVA’s were used to analyze the ERP indices of response inhibition with Inhibition and Picture as within-subjects variables and trait anger as covariate for the N2 and P3 component, and with accuracy (Correct Go vs. Incorrect NoGo) as within-subjects variables for the ERN and Pe component. In order to prevent the covariate from altering the main effect of the repeated measure while running the ANCOVA, trait anger scores were centered using the method of Delaney and Maxwell (1981), i.e. using the individual Trait Anger score minus the mean Trait Anger score of all participants so that the mean of the covariate trait anger was by definition equal to zero. Pearson correlation coefficients were calculated for the accuracy on the Go/NoGo task and ERP indices on the one hand and trait anger on the other hand.

## Results

### Behavioral Data: Trait Anger and Performance on Inhibition

Table 2 presents the accuracy and reaction time data on the Go/NoGo task. On average participants were less accurate on NoGo trials than on Go trials (77.7 vs. 95.5% respectively), *F*(1,35) = 71.82, *p* < .001, $\eta _{p}^{2}$ = .67. With regard to the reaction time data, no differences were found between Anger-Related Go trials (*M* = 371.78, *SD* = 83.28) and Neutral Go trials (*M* = 372.93, *SD* = 77.04), *F* < 1.

**Table 2:** Accuracy rates (in proportions) and reaction times (in ms) on the anger related Go/NoGo task

	*M*	*SD*
Acc Go Agr	.95	.07
Acc Go Neutr	.96	.06
Acc NoGo Agr	.76	.17
Acc NoGo Neutr	.79	.14
RT (ms) Go Agr	372	83
RT (ms) Go Neutr	373	77

One goal of this study was to examine whether trait anger was negatively associated with performance on inhibition (i.e., the NoGo trials), in particular for the anger-related pictures. Results show that on average the accuracy of responding on NoGo trials did not differ for anger-related pictures and neutral pictures, *F*(1,34) = 3.38, *p* = .075, $\eta _{p}^{2}$ = .09. Importantly, trait anger was significantly associated with the accuracy of responding, *F*(1,34) = 7.89, *p* = .008, $\eta _{p}^{2}$ = .19. Moreover, a significant trait anger × picture interaction, *F*(1,34) = 4.50, *p* = .041, $\eta _{p}^{2}$ = .12, indicated that higher scores on trait anger were accompanied by even lower accuracy for anger-related pictures than for neutral pictures. To follow-up on these results, correlations between the Trait anger scale and the accuracy rates on the NoGo trials were calculated. As expected, higher trait anger scores were associated with lower accuracy on both Neutral (*r* = − .36, *p* = .034) and Anger-Related NoGo trials (*r* = − .47, *p* = .004). Moreover, accuracy was indeed lower for anger-related pictures than for neutral pictures.

To examine whether these effects of trait anger were specific for NoGo trials, we additionally explored whether trait anger influenced accuracy of responding on Go trials in a similar fashion. Results revealed that, similar to the accuracy of responding on NoGo trials, accuracy for the Go trials on average did not differ for anger-related pictures and neutral pictures, *F*(1,34) = 3.78, *p* = .060, $\eta _{p}^{2}$ = .10. More importantly, trait anger was not related to the accuracy of responding on Go trails and neither showed an interaction with Picture content, both *F*s < 1.

### ERP Data: Trait Anger and ERP Indices of Response Inhibition

Another goal of this study was to investigate whether higher scores on trait anger were accompanied by decreased N2, P3, ERN, and Pe amplitudes and whether this effect was more pronounced for anger-Related pictures.

**Fig. 1:**
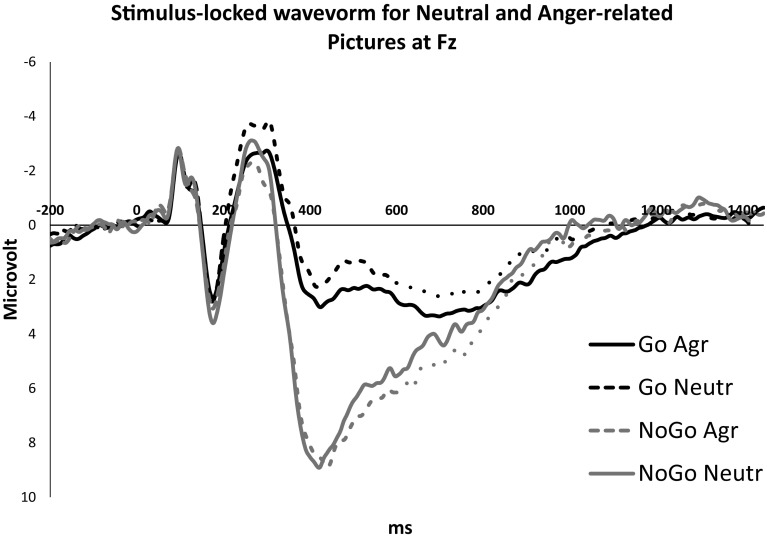
Grand-average stimulus-locked wavevorms for neutral and anger-related pictures at Fz for correct Go and NoGo trials

Figure 1 depicts the grand-average stimulus locked waveforms for neutral and anger-related pictures at Fz for both correct Go and NoGo trials. Contrary to expectation, the N2 amplitudes on Go and NoGo trials in general did not differ, *F*(1, 33) = 2.61, *p* = .116, $\eta _{p}^{2}$ = .07, and there was also no significant picture × inhibition interaction, *F* < 1. We did find a main effect of Picture on the N2 component, *F*(1, 33) = 8.10, *p* = .008, $\eta _{p}^{2}$ = .20, showing a less negative wave on the N2 component for the anger-related pictures compared to neutral pictures. Importantly, trait anger was not related to the N2 amplitude, *F* < 1. As a general inhibition effect on the N2 amplitude was not found, no follow up analyses were conducted regarding the N2 component.

**Fig. 2:**
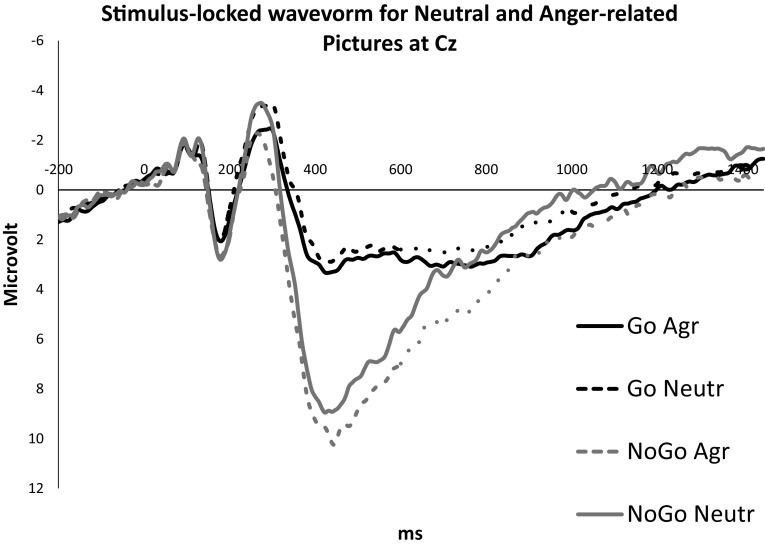
Grand-average stimulus-locked wavevorms for neutral and anger-related pictures at Cz for correct Go and NoGo trials

The P3 amplitudes for anger-related and neutral pictures at Cz for correct Go and NoGo trials can be seen in Fig. 2. As expected, the P3 amplitudes were generally larger for NoGo trials than for Go trials, *F*(1,33) = 67.46, *p* < .001, $\eta _{p}^{2}$ = .67. However, the P3 amplitudes did not significantly differ for anger-related trials and neutral trials, *F*(1,33) = 3.17, *p* = .084, $\eta _{p}^{2}$ = .09. Moreover, the inhibition × picture interaction was not significant, *F*(1,33) = 1.00, *p* = .323, $\eta _{p}^{2}$ = .03. Importantly, trait anger was not related to the P3 component, *F*(1,33) = 3.72, *p* = .062, $\eta _{p}^{2}$ = .10, and no significant interaction effects of trait anger were found, all *F*s < 1. In sum, these results seem to suggest that trait anger is not associated with the inhibition associated P3 amplitude.

**Fig. 3:**
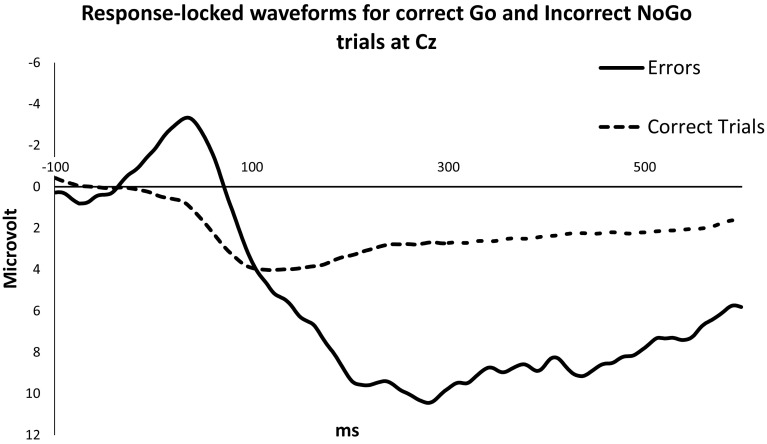
Grand-average response-locked wavevorms for neutral and anger-related pictures at Cz for correct Go and incorrect NoGo trials

The grand-average response-locked waveforms at Cz for correct Go and incorrect NoGo trials are depicted in Fig. 3. According to expectation, the ERN was larger for errors than for correct responses, *F*(1,30) = 20.27, *p* < .001, $\eta _{p}^{2}$ = .40. There was no main effect of trait anger, *F*(1,30) = 2.04, *p* = .164, $\eta _{p}^{2}$ = .06. In line with our hypothesis, we did find a significant Accuracy x Trait anger interaction, *F*(1,30) = 4.75, *p* = .037, $\eta _{p}^{2}$ = .14, indicating that higher scores on trait anger were associated with reduced ERN amplitudes.^1^

Similar to the ERN, the Pe amplitudes were larger for errors than for correct responses, *F*(1,30) = 22.82, *p* < .001, $\eta _{p}^{2}$ = .43 (see Fig. 3). However, no main or interaction effect of trait anger was found for the Pe, both *p*s > .80, indicating that trait anger was not significantly related to the Pe.

## Discussion

The main goal of the present study was to investigate whether individual differences in trait anger in violent offenders are associated with individual differences in anger-primed inhibitory control using behavioral and electrophysiological measures of response inhibition. On the behavioral level, we expected higher scores on trait anger to be associated with more mistakes on the infrequent NoGo stimuli, in particular for the Anger-Related pictures. Consistent with these predictions, higher scores on trait anger were accompanied by lower accuracy on NoGo trials, and even lower accuracy for Anger-Related pictures. On the neurophysiological level, we expected higher scores on trait anger to be associated with reduced NoGo N2 and P3 amplitudes and with reduced ERN and Pe amplitudes. Contrary to our expectations, analyses of the stimulus locked ERP waves revealed that both the N2 and the P3 components were not related to trait anger. With respect to the response locked ERP waves, we did find a significant inverse relation between trait anger and the ERN amplitudes for incorrect NoGo trials, but not between trait anger and the Pe.

Previous behavioral studies have shown that inhibitory control is related to trait anger and reactive aggression, particularly in hostile contexts (Wilkowski et al. 2010; Wilkowski and Robinson 2008a; Wilkowski 2011). Most of these studies were conducted within healthy subjects high on trait anger and used a flanker task, leaving the question to what extent these results could be generalized to more pathological samples, such as violent offenders, and different measures of cognitive control. The current study shows that also in violent forensic psychiatric patients inhibitory control is inversely related to trait anger. Moreover, we found the same inverse relation among trait anger and anger-primed inhibitory control with a new measure of response inhibition: an affective Go/NoTask. Hence, the current study adds further support to the integrative cognitive model of trait anger and reactive aggression (Wilkowski and Robinson 2008b, 2010) and converges with theories emphasizing the situation-specificity of self-control (Kerns et al. 2004). Finally, results are also in line with other models highlighting the importance of self-control with respect to aggression and violence, such as the I3 theory (Finkel 2007) and the General Aggression Model (DeWall et al. 2011).

Analyses of the stimulus locked ERP waves revealed that both the N2 and the P3 components were not related to trait anger. Given that individual differences in trait anger in violent offenders are related to deficits in impulse control on a behavioral level, these relations are not easily explained by deficits in neural processes involved in response inhibition. The absence of an inverse relation between trait anger and the N2 and P3 in the current study seems in line with the results of the study of Liu et al. (2014), who also found no differences on the N2 and P3 components between low and high trait anger healthy participants on NoGo trials in a visual Go/NoGo paradigm, and with results from Munro et al. (2007) who found that violent offenders with psychopathy made more errors of commission on NoGo trials, but did not differ from healthy controls regarding the N2 and P3 components. On the contrary, our results are not in line with those of Chen et al. (2005), who found lower N2 amplitudes in impulsive-violent offenders compared to matched offenders that did not commit offenses of an impulsive-violent nature. These contrasting findings can possibly be ascribed to differences in the difficulty level and nature of the tasks that were used as well as by differences in the subtypes of violent offenders that were included. For instance, although trait anger is associated with the degree of clinical significant anger problems (DiGiuseppe and Tafrate 2010), Davey et al. (2005) showed that some violent offenders may be characterized by ‘over-control’ of anger whereas others may be better characterized by under-controlled anger. Furthermore, there are indications that especially impulsive violent offenders low in psychopathy demonstrate reduced NoGo N2 effects whereas violent offenders high in psychopathy do not (Munro et al. 2007; but also see; Kiehl et al. 2000). Another possible explanation lies in the difference between the numbers of reactive and instrumental aggressors in the various research samples. Whereas reactive aggression is closely related to impulsivity, instrumental aggression is assumed to be more cold-blooded and thus less impulsive (Barratt et al. 1999). As the study by Chen et al. (2005) in a sample of impulsive violent offenders, is the only one that found reduced N2 effects it seems likely that especially impulsive aggressive behaviors are related to neurophysiological indices of response inhibition. Altogether, we propose that whereas reduced N2 and P3 amplitudes may be present in violent offenders, they are not related to the severity of problematic anger and are perhaps better explained by the degree of impulsive aggressive behaviors exhibited. Future studies that take these subtypes into account should be conducted in order to test this idea. In doing so, these studies might benefit from including affective tasks instead of affective-neutral tasks as we found different results for anger-primed inhibitory control compared to neutral inhibitory control.

With respect to the response locked ERP waves, we did find a significant inverse relation between trait anger and the ERN amplitudes, but not between trait anger and the Pe. Whereas the ERN is proposed to reflect the automatic stage of error detection or conflict monitoring (Bernstein et al. 1995; Yeung et al. 2004) or may also reflect attentional control to unexpected events (Van Noordt et al. 2015), the Pe appears to reflect the more conscious reflection or awareness of the motivational salience of an error (Overbeek et al. 2005). Accordingly, the current study results suggest that individual differences in trait anger in violent offenders are related to individual differences in deficits in automatic error-processing, but not to deficits in more conscious stages of error-processing. It seems likely that these deficits in automatic error-processing contribute to the continuation of impulsive angry behavior in dipositionally angry people despite its negative consequences. However, it should be noted that the current study design does not allow drawing conclusions on causality. For example, it might also be that reduced error-processing predisposes individuals to impulsive behaviors, including aggressive behaviors, or that impulsive behaviors lead to less error-processing in the long run. Moreover, the relation between trait anger and reduced error processing may also be explained by an underlying externalizing factor as previous studies have shown impaired error monitoring to be associated with externalizing psychopathology (Hall et al. 2007; Olvet and Hajcak 2008).

An important prerequisite for the current study was that the Go/NoGo task worked as intended given that we used a clinical sample as well as that the neurological indices reflected the proposed underlying processes. With respect to accuracy of responding, task performance was similar to the pattern that is usually obtained in Go/NoGo tasks, i.e. lower accuracy on NoGo trials than on Go trials (71.4 vs. 95.6% respectively). Moreover, both the ERN and the Pe were significantly enhanced on incorrect NoGo trials compared to correct Go trials converging with the view these ERP’s reflect error-processing. Interestingly, the P3 was significantly enhanced on NoGo trials compared to Go trials whereas the N2 was not. A possibility may be that the N2 does reflect inhibitory processing, but was not more pronounced on NoGo trials in the current study because we used a clinical violent sample who are proposed to have weakened NoGo N2 amplitudes and intact P3 amplitudes (Davidson 2000). In line with this reasoning the N2 amplitudes were smaller for anger-related pictures than for neutral pictures, which could indicate that our violent offenders recruited less inhibitory control in hostile contexts. Moreover, we did find the general N2 Nogo effect in undergraduate students using the same task (Lievaart et al. 2016). In short, we can conclude that the patients in the current study performed the task reasonably well and that the data obtained from this study are reliable.

While our study benefitted from the use of a forensic psychiatric sample and the inclusion of affective cognitive task allowing to examine neutral and anger-primed inhibitory control, the present study has some limitations that are important to address. One obvious limitation is the absence of a control group. However, our main focus was to investigate whether individual differences in impulse control are associated with individual differences in trait anger. Second, one could question the use of self-report data in forensic samples as forensic patients are sometimes inclined to minimize their experience of anger and aggression (McEwan et al. 2009). However, even if this would be the case in the current study this would most likely lead to an underestimation of the actual effects and therefore have little impact on our main conclusions. Another limitation of the study is that we did not measure current medication use in the research sample. Medication might have influenced the results of the present study. An additional limitation of the study was that we only used angry and neutral stimuli. This does not allow to see whether the present results are specific or could possibly be attributable to emotional stimuli in general. In addition, we used a fixed order of picture presentation (always neutral first to avoid carry-over effects). However, the fixed order could have resulted in unwanted order-effects. Finally, our sample was relatively small.

In conclusion, the results of the current study showed that individual differences in trait anger in forensic psychiatric patients can be related to individual differences in impulse control on a behavioral level and reduced automatic error-detection on a neurophysiological level. However, the hypothesis that higher trait anger is associated with lower N2 and P3 amplitudes could not be confirmed. These results suggest that high trait anger forensic patients have difficulties with inhibitory control and error detection, which could explain the initiation and continuation of angry impulsive behavior despite its negative consequences.

The current study is of a relatively fundamental nature and therefore may not have immediate clinical relevance. Although it may take some time for these fundamental findings to be translated into clinical practice, this study does, however, inform indirectly about practical implications next to providing important theoretical implications. For instance, EEG might prove useful as a diagnostic tool in the treatment of dysfunctional anger in the near future. Although more information is needed on the predictive validity, the ERN and the Pe show excellent psychometric properties and can be measured using relatively few trials (Hofmann et al. 2012; Olvet and Hajcak 2009; Rietdijk et al. 2014). As such, they may inform about individuals at risk for relapsing into negative behavior despite their negative consequences and may be used to predict efficacy of and dropout from anger management therapies. Previous studies, for instance, have shown that cognitive control deficits may indicate fewer capacities to recognize problematic behavior, reduced motivation for treatment as well as dropout from therapy in substance abusers (Ersche and Sahakian 2007; Severtson et al. 2010). Finally, direct training of brain regions related to cognitive control and error processing, such as the anterior cingulate cortex, inferior frontal gyrus and dorsolateral prefrontal cortex, via neurofeedback techniques, deep brain stimulation or via repetitive transmagnetic stimulation can inform about the causal relation between neurocognitive processes and trait anger and may be used for treatment in order to reduce anger. Recent studies have also shown that cognitive bias modification paradigms targeting cognitive control, may be effective in reducing anger or aggression (Wilkowski et al. 2015).

## Footnotes

1 Importantly, peak measures yielded similar results. Moreover, the significant accuracy × trait anger interaction was retained when both Fz and Cz were included in the repeated measures ANOVA.
